# A novel methodological framework for predicting and mapping agriculture-related soil attributes using Euclidean distance, regular grids, and machine learning algorithms

**DOI:** 10.1371/journal.pone.0343624

**Published:** 2026-05-11

**Authors:** Gustavo Vieira Veloso, Danilo César de Mello, Elpídio Inácio Fernandes-Filho, Cristiano Marcelo Pereira de Souza, Lucas Augusto Pereira da Silva, Mario Marcos Espirito Santo, Gustavo Mattos Vasques, Maurício Rizzato Coelho, José A. M. Demattê

**Affiliations:** 1 Department of Soil Science, Federal University of Viçosa, Viçosa, MG, Brazil; 2 Department of Soil Science, “Luiz de Queiroz” College of Agriculture, University of São Paulo, Piracicaba, SP, Brazil; 3 Geology Board, Federal University of the São Francisco Valley - Senhor do Bonfim Campus R. Tomás Guimarães, Sr. do Bonfim, BA, Brazil; 4 Geography Department, Campus III – Guarabira, State University of Paraíba (UEPB), Guarabira, PB, Brazil; 5 Department of General Biology, State University of Montes Claros, Montes Claros, MG, Brazil; 6 Embrapa Soils, Rio de Janeiro, RJ, Brazil; GMR Institute of Technology, INDIA

## Abstract

Recent advances in statistical and machine learning (ML) methods have improved the prediction of soil attributes at fine spatial scales, yet the comparative performance and reliability of these techniques remain unclear. This study compared Ordinary Kriging (OK), Inverse Distance Weighting (IDW), and ML algorithms in predicting and spatializing soil attributes, while also evaluating prediction uncertainty and computational processing time. Conducted in Minas Gerais State (Brazil), the analysis used Euclidean distance based predictors derived from X-Y coordinates and regular grids with 5, 7, and 10 divisions. Soil attribute maps (CEC, phosphorus, sand, and clay) were generated using OK, IDW, Random Forest (RF), Cubist, Support Vector Machine (SVM), and Earth. Model performance was assessed using R^2^, RMSE, MAE, and the coefficient of variation. IDW and OK showed the lowest predictive accuracy (R^2^ = 0.52–0.58), whereas ML methods, especially RF and SVM achieved superior performance (R^2^ = 0.62–0.70). Among ML algorithms, Earth performed worst, while RF produced the highest accuracy for all attributes except sand, for which SVM performed best. Processing time was shortest for IDW, followed by OK; among ML models, Earth was fastest, followed by RF, SVM, and Cubist. Larger regular grids improved ML prediction and spatialization but increased computational cost. ML methods thus outperform traditional geostatistical interpolators, benefiting from the use of numerous covariates and flexible algorithmic structures, although requiring greater computational time. These findings demonstrate the robustness and practical potential of ML approaches for soil attribute mapping.

## Introduction

Soils constitute a highly heterogeneous triphasic system whose mineralogical, biological, and physicochemical attributes vary across space and time due to the combined action of soil-forming factors [1,2]. Understanding this spatial variability is fundamental for interpreting soil-environment interactions [3], guiding sustainable management [4], and designing sampling strategies in environmental and agricultural studies [5]. The growing demand for detailed soil information in precision agriculture and environmental modeling [6] has intensified the need for robust approaches to quantify variability.

Traditional soil characterization relies on laboratory analyses and field surveys [7], but these procedures are time-consuming, costly, and spatially limited, as samples represent point observations that must be extrapolated over entire landscapes [8]. Addressing this challenge requires integrating remote sensing, geotechnologies, and numerical spatialization methods [9].

Digital soil mapping frameworks provide tools to transform point data into continuous spatial predictions [10,11]. Among these, inverse distance weighting (IDW) and ordinary kriging (OK) are widely applied [12,13]. However, their relative accuracy remains inconsistent across studies [14,15] because both methods rely on assumptions about spatial dependence that may not hold in complex pedological environments. OK requires variogram modeling and dense sampling to characterize spatial autocorrelation, which is often impractical [11], while IDW applies distance-based weighting without accounting for underlying environmental controls and does not estimate prediction uncertainty [16,17]. Both methods may also generate unrealistic “bulls-eye” artifacts [18].

Machine learning (ML) techniques have emerged as powerful alternatives for predicting soil attributes influenced by nonlinear interactions among soil-forming factors [2]. ML has been successfully applied to estimate SOC, texture, pH, CEC, macro- and micronutrients, heavy metals, and other soil properties [19–23]; Jafarzadeh et al., 2016; Gao et al., 2019). Common algorithms include Random Forest, Support Vector Machines, Artificial Neural Networks, K-Nearest Neighbors, and Cubist models. Unlike OK and IDW, ML does not require spatial dependence and can incorporate a large number of covariates, providing both high predictive performance and insights into variable importance [24,25].

However, ML generally demands greater computational effort and may rely on georeferenced inputs only through their use as covariates (e.g., SCORPAN’s “N” factor). Despite advances, few studies evaluate the combined use of ML with Euclidean-grid covariates derived from X-Y coordinates to overcome sampling density limitations. Early evidence suggests that ML can match or exceed the performance of OK and IDW for spatializing soil properties [26,27].

The incorporation of Euclidean distance as a spatial covariate in our framework is grounded in its capacity to represent the geometric proximity between sampling points in a continuous, isotropic space, an essential property for soil systems in which gradual spatial transitions often occur over short geographic distances. Unlike variogram-based dependence measures, which require stationarity, substantial sampling density, and explicit modeling of spatial autocorrelation, Euclidean distance provides a model-agnostic and computationally efficient descriptor of spatial structure that can be directly integrated into machine learning algorithms [26]. Furthermore, Euclidean metrics have been widely employed in environmental modeling and spatial ecology to represent spatial gradients when autocorrelation is weak, heterogeneous, or difficult to parameterize [28]. Compared with alternative distance metrics, such as Manhattan distance, Minkowski distance, or kernel-based measures, Euclidean distance preserves geometric interpretability, avoids anisotropy assumptions, and performs consistently across regular grid systems [26,29]. Its use in this study is not intended to replace geostatistical dependence models but rather to offer a flexible spatial descriptor that machine learning algorithms can exploit to capture nonlinear spatial patterns that are otherwise inaccessible through traditional interpolators [30]. This theoretical and practical rationale underpins the importance of Euclidean distance as a core component of our proposed workflow.

Despite the extensive adoption of machine learning and geostatistical interpolators in digital soil mapping, current frameworks do not integrate spatial information into machine learning models through explicit Euclidean distance–based covariates combined with multiple regular grid systems, nor do they evaluate how distinct grid configurations influence predictive performance and uncertainty [31]. In contrast to previous studies that rely on single-grid or purely covariate-based approaches, our methodology systematically tests alternative grid structures to optimize spatial representation within machine learning workflows. Moreover, we introduce a rigorous repeated hold-out validation protocol that quantifies both predictive accuracy and spatial uncertainty, providing a more robust evaluation than conventional cross-validation strategies typically adopted in soil mapping. A further innovation of this work is the comprehensive benchmarking of computational processing times across diverse machine learning algorithms and geostatistical methods, addressing practical constraints of scalability and efficiency that remain largely overlooked in the literature. By assessing multiple predictive models within different spatial frameworks, our study offers new insights into algorithmic behavior, strengths, limitations, and suitability for specific soil attributes. Altogether, this integrated framework represents a substantive methodological advance, bridging existing gaps in digital soil mapping and delivering a versatile, scalable, and reliable approach for improving spatial prediction of soil properties.

In addition to proposing an integrated Euclidean–grid machine learning framework, this study explicitly benchmarks its performance against established baseline approaches. These include (i) traditional geostatistical interpolators widely used in digital soil mapping, Ordinary Kriging (OK) and Inverse Distance Weighting (IDW), and (ii) standalone machine learning models that rely solely on X-Y coordinates without spatial covariate enhancement. Such baselines provide a rigorous reference for evaluating performance gains, allowing us to isolate the contribution of grid-based Euclidean covariates from both classical geostatistical assumptions and standard ML predictive behavior. In this context, the present study systematically assesses machine learning algorithms for predicting and mapping soil attributes, comparing their performance, uncertainty, and computational efficiency against traditional approaches such as IDW and ordinary kriging.

As previously described, the research aimed to: *i)* Insert spatial information using various regular grids into machine learning (ML) algorithms and assess model performance for soil attribute modeling; *ii)* compare the performance of ML algorithms with interpolators that solely utilize spatial information; *iii)* evaluate and compare the computational processing time of spatialization methods. Our main hypotheses are: *1)* Inserting spatial information into ML algorithms increases performance in the modeling process; 2*)* ML algorithms achieve superior performances than interpolators that operate only with spatial information; 3*)* The modelling process by ML algorithms require more time consuming;

This comparison and evaluation are crucial for soil science and geoscience community because, if we are only able to produce an average spatialized soil attributes map related to the overall uncertainty (i.e., cross-validation error), we may provide end-users with soil attributes maps that are, completely unreliable and unusable for agriculture and environmental practices.

## Materials and methods

### Study area

**Fig 1:**
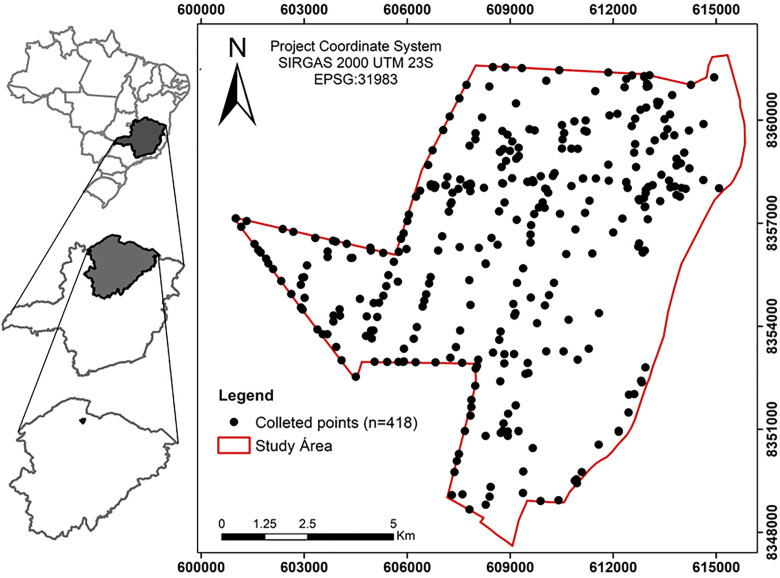
Study area and collected points. Source: Maps were produced using territorial mesh data from IBGE (https://www.ibge.gov.br/geociencias/organizacao-do-territorio/malhas-territoriais/15774-malhas.html) and data from an open-access SciELO publication (https://www.scielo.br/j/pab/a/jgVjx6jHPzWjqPLqMgYttzJ/?format=pdf&lang=en). Data are available under the Creative Commons Attribution License (CC BY 4.0) or equivalent, with proper attribution.

The study area is located in the North of Minas Gerais State, Brazil, from 44.06179 ºW to 43.92339 ºW longitude and 14.94412 ºS to 14.81475 ºS latitude, in the Conservation Unit “Parque Estadual da Mata Seca” with an area of 102 km^2^ (Fig 1). The vegetation cover of the area is naturally complex, composed of distinct vegetation formations, predominantly deciduous, and it is inserted in the wide transitional range between the “Cerrado”, the “Caatinga” and Atlantic Forest biomes [32].

The main soil types, according to [33] classified in the area are: Dystric Xanthic and Rhodic Ferralsols, Xanthic Cambisols, Dystric Gleysols, Haplic Phaozem and Dystric Fluvisols [34]. Elevations range from 434 to 523m in a flat relief. The main lithological formations are: The “Urucuia” Group, which is mainly composed of sandstone from the Cretaceous Period; The “Bambuí” Group, which are composed of siltstone, shale, marl, and limestone, from the Neoproterozoic Era [34].

The region’s climate is semi-arid, classified as transitional between the typologies Aw and BSw following the Köppen classification [35]. The average temperature is 25 °C, while the mean annual precipitation varies between 600 and 828 mm [34].

### Sample database

The soil sample database used in this study was obtained from the Brazilian Agricultural Research Corporation (EMBRAPA) [34]. A total of 418 soil samples were collected across the study area following a stratified sampling scheme designed to capture the main lithological units, geomorphological domains, and soil types. This strategy ensured that the dataset represented the environmental heterogeneity of the region and reduced potential spatial clustering effects. Samples were taken from the 0–20 cm soil layer using a standardized auger protocol [36], and all sampling points were georeferenced to allow spatial analysis and integration with pedogeophysical and environmental covariates (Fig 1).

The augering protocol was defined based on landscape (relief) observations and supported by pre-existing cartographic resources, including soil maps, planialtimetric charts, and the digital elevation model. Following the approach described by Demattê and da Silva Terra (2014), sampling points were established using hand augers (boreholes), ensuring consistent depth, representativeness, and operational standardization across the entire study area. To ensure representativeness, sampling locations were distributed proportionally across the major soil-landscape compartments, including areas with contrasting parent materials, slope positions, and surface conditions. This distribution provides a balanced dataset that reflects the pedogenetic and environmental gradients of the region.

For each sample, traditional physico-chemical analyses were conducted according to the standardized methodologies described in [37]. The following soil attributes were used in this study: clay content, sand content, available phosphorus (P), and cation exchange capacity (CEC). Clay and sand fractions were determined by the pipette method, following chemical dispersion with NaOH and subsequent particle sedimentation. Available phosphorus (P) was quantified using the Mehlich-1 extraction followed by colorimetric determination. Cation exchange capacity (CEC) at pH 7.0 was obtained by cation saturation with ammonium acetate, extraction, and subsequent quantification of exchangeable bases. These procedures follow the official analytical protocols established by EMBRAPA for soil characterization in Brazil. Detailed analytical protocols for each variable are provided in [37].

### Standard grid covariates used in machine learning

The regular grids used in this study were constructed based on the fixed spatial boundary of the study area. Grid resolution was varied by increasing the number of subdivisions along the X and Y axes (e.g., 2, 5, 7, and 10 divisions), which proportionally decreases the Euclidean distances between grid nodes and effectively increases spatial sampling density. This systematic variation functions as a sensitivity analysis, enabling us to assess how machine learning algorithms respond to different spatial resolutions and determining the extent to which grid granularity influences predictive accuracy and uncertainty. Each grid configuration was evaluated independently across all models to ensure a robust comparison of spatial resolution effects.

**Fig 2:**
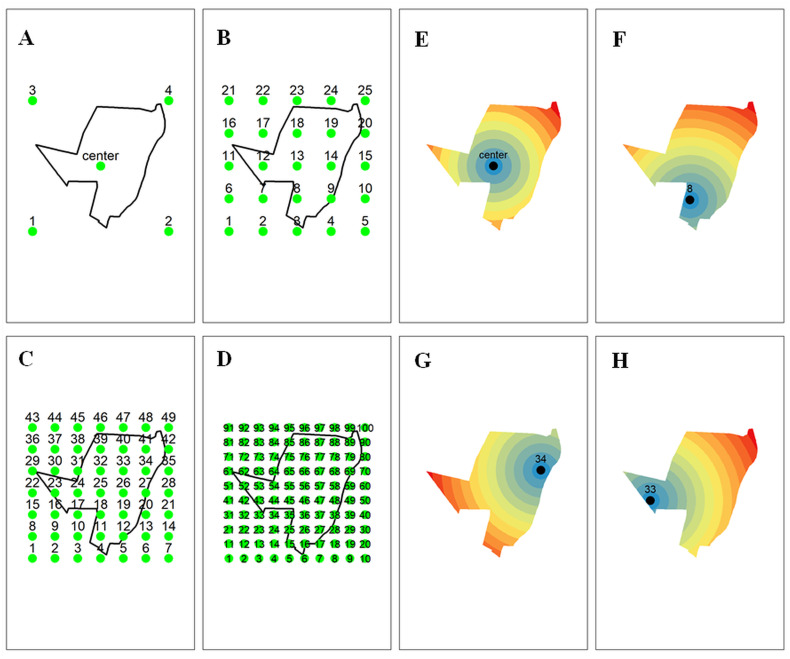
Study area and grid systems. A: 2 x 2 grid + area center point + X, Y (7 covariates); B: 5 x 5 grid + X, Y (27 covariates); C: 7 x 7 grid + X, Y (51 covariates); D: 10 x 10 grids + X, Y (102 covariates). Figs 2E and 2H illustrate examples of the derived covariates computed for each sample point depicted in Figs 2A to 2D.

The sample database (2.2 section) was added covariates created from the Euclidean distance of regular grid points (Fig 2). The covariates are created from equidistant points more geographic coordinates (X, Y), with their creation exclusively relying on the analysis of the study area. No additional environmental, physical, chemical, or biological information from the area is required. To construct the sample database (Section 2.2), we incorporated covariates derived from the Euclidean distance between each sampling location and regularly spaced grid nodes distributed across the study area (Fig 2). These covariates were generated exclusively from equidistant grid points and the original geographic coordinates (X and Y), relying solely on the spatial configuration of the study area and requiring no additional environmental, physical, chemical, or biological information.

Five grid systems were evaluated, each defined by a regular arrangement of points distributed horizontally and vertically according to the maximum extent of the study area along both axes:

(i) X and Y coordinates only (2 covariates);
(ii) X and Y combined with a 2 × 2 grid and the centroid of the study area (7 covariates) (Fig 2A and 2E), equivalent to the system proposed by Behrens et al. (2018);
(iii) X and Y combined with a 5 × 5 grid (27 covariates) (Fig 2B and 2F);
(iv) X and Y combined with a 7 × 7 grid (51 covariates) (Fig 2C and 2G);
(v) X and Y combined with a 10 × 10 grid (102 covariates) (Fig 2D and 2H).

For all grid systems, Euclidean distances between each grid node and all pixels within the study area were calculated in meters, producing continuous distance layers used as covariates in the machine-learning models. The resulting maps had an approximate spatial resolution of 30.43 meters per pixel.

The generation of the grid systems and the automated computation of Euclidean distances were performed using the *getdist_rectangle* function from the **labgeo** package (Fernandes Filho, 2019), which implements an adaptation of the methodology proposed by Behrens et al. (2018) for constructing regular grid structures.

This methodology represents an evolution of the approach outlined by Behrens et al. (2018), where they utilized the corners of a rectangle surrounding the sample set (C1, C2, C3, C4). The grid X, y + 2 x 2 grid + area center system is equivalent to that used by this author (Fig 2A and 2E). The degree systems test were: equidistant points with 5, 7 or 10 vertical and horizontal points respectively together with the coordinates X and Y (Fig 2B, 2C, 2D, 2F, 2G and 2H). We adapted the methodology described by [26] to create the grid systems. The grid systems used were: equidistant points, with five (5,7 or 10) points vertically and five (5, 7 or 10) horizontally. These points took into account the study area limits vertically and horizontally. The definition of grid was calculated at Euclidean distances in meters from the grid points (Fig 2E, 2F, 2G and 2H). Finally, the grids and calculation of the Euclidean distance were automated performed the *getdist_rectangle* function from the *labgeo* package [38].

With the grid, coordinate (X, Y) and the sample database, we used the *stack* and *extract* function of the *raster* package [39] in the *R* software [40], which comprises the stacking of covariates for each of the grid systems tested and the determination of covariate values at the sampling points. Then, the final database was created for training and spatialization by ML algorithms.

### Modelling by machine learning algorithms

**Fig 3:**
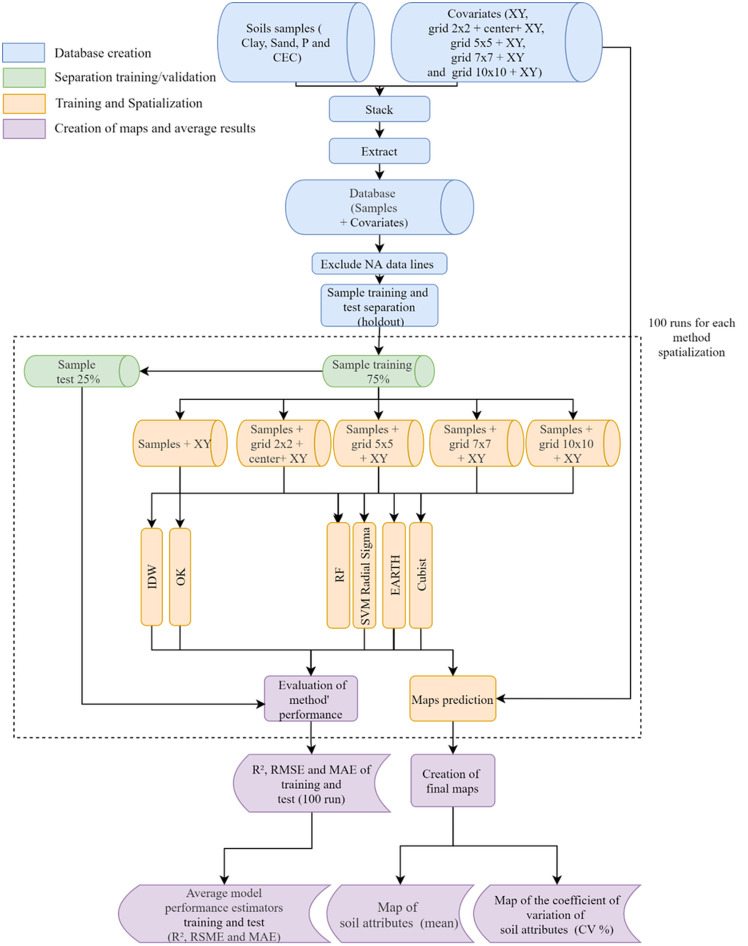
Methodological flowchart showing the sequence of methodologies applied for soil attributes prediction and spatialization.

The modeling workflow is illustrated in Fig 3. Data were initially partitioned using a hold-out approach, in which 75% of the samples were used for training and 25% for testing [41]. This partitioning was implemented with the createDataPartition function from the **caret** package, which performs a stratified split to maintain the distribution of the response variable across subsets. This training/testing procedure was repeated 100 times (repeated hold-out), enabling the estimation of prediction variability arising from different random splits. The same training and testing sets were used for all algorithms in each iteration, and the identical sampling scheme was applied to IDW and OK.

To ensure a fair and robust comparison with baseline methods, both OK and IDW were implemented using the exact same 100-round repeated hold-out scheme applied to machine learning algorithms. Additionally, to provide a baseline ML reference, models were also trained using only the original X-Y coordinates, without additional Euclidean-distance covariates. This ensures that performance differences can be attributed directly to the proposed spatial covariate framework rather than to unequal sampling strategies or differences in model configuration.

Model training followed the same procedure for all algorithms and grid configurations. Hyperparameter optimization was carried out within the training set using repeated cross-validation (repeatedcv) with 10 folds and 10 repetitions, as implemented in the train function [42]. This resampling strategy systematically partitions the training data, fits the model to each fold, and repeats the process across the specified iterations, providing a stable estimate of predictive performance during tuning.

Hyperparameter tuning was performed independently for each algorithm using the *train* function of the caret package, which conducts a systematic grid search over user-defined parameter ranges. We evaluated four algorithms: Cubist, Multivariate Adaptive Regression Splines (Earth), Random Forest (RF), and Support Vector Machine with Radial Sigma kernel (SVM). The following hyperparameters were tuned: committee size and number of neighbors for Cubist; nprune and polynomial degree for Earth; mtry for RF; and sigma and C for SVM. Candidate values were generated automatically by the train function using the user-defined search range (grid of 8 equidistant values per parameter). The same tuning ranges were applied across all soil attributes to ensure methodological consistency among models. To avoid overfitting, model training and hyperparameter selection were performed using repeated 10-fold cross-validation (ten repetitions), and the optimal configuration for each algorithm was selected based on the lowest mean absolute error (MAE) averaged across all resampling iterations. A complete description of the hyperparameters for each model is available in the caret documentation (https://topepo.github.io/caret/train-models-by-tag.html).

The Random Forest (RF) algorithm combines qualitative and quantitative data to create multiple random trees. Each tree derives from a randomly chosen subset of sample data and a subset of predictors, which this algorithm estimates errors using a subset of the database that is not used in the tree construction data [43]. The standard size of this subset used to estimate the error is approximately one-third of the database. Three parameters that can be modified to improve the performance of the RF model: the number of trees in the forest (*ntree*); the minimum number of data points in each terminal node (*nodesize*) and; the number of features tried at each node (*mtry*) [44]. Only the *mtry* parameter will show significant changes to the prediction when modified [41,45]. The caret package optimizes it leaving *nodesize* and stable *ntrees* with values of 5 and 500,respectively [41,44].

The Cubist model is a non-conventional rule-based decision tree regression, being an extension of the M.5 algorithm [46]. It creates a decision tree where in the model inputs (sheets), internal rules based on a multivariate linear model are used, which data and subsets are partitioned [47,48]. In addition, Cubist has linear regressions in the intermediate steps. Its quality is to be able to eliminate some outliers when applying the regression models [47]. Cubist calibration parameters are committees and neighbors. Committees are used for more balanced model constants and values greater than one are used. The *neighbor’s* parameter is used to fix the internal rules.

The Support Vector Machine (SVM) was developed by [49], which is considered a classification algorithm. It is based on statistical learning theory that seeks to minimize errors in the model structure by minimizing the confidence interval. To solve non-linear problems, data are projected in a hyperspace where it is possible to separate them linearly [50]. Later, SVM creates a hyperplane with margins of known size, where errors are small. In this way, a good generalization ability is obtained. The algorithm minimizes these margins and increases the model’s precision [51]. The Support Vector Machines with Radial Basis Function Kernel has as optimization parameters a penalty (cost) which controls the trade-off between margin and training errors and the kernel width (Sigma) which controls the degree of nonlinearity of the model.

The Multivariate Adaptive Regression (Earth) is a nonparametric linear regression method that has the partitioning of the data. The data set are separated into segments by parts (spline), where linear regressions with different slopes are calculated [52]. The starting and ending points of the segments are called nodes, and the algorithm is capable of evaluating the importance of the variables in each segment and is also the position of the nodes. Its quality does not have any specific assumptions between functions that relate input and output variables [53].

### Interpolation and uncertain by ML algorithms

The training model was used in modelling for each pixel/cell in the study area. For this, 100 maps were generated for each attribute (clay, sand, CEC and P), ML algorithm and tested covariates (grids). These maps were used in creating the final maps composed of the mean value of 100 maps for each pixel. For the study of prediction/mapping uncertainty, the coefficient of variation map (CV% = standard deviation/mean) of each pixel of the tested grid and algorithm and, then soil attributes were generated.

### Interpolation by IDW and OK

Spatialization of soil attributes follows a methodology used by Machine Learning’s techniques for use of IDW and OK interpolators, mainly in the separation of the training group and the 100 rounds process.

Inverse Distance Weighted interpolation assumes that the closer things are to each other, the greater similarities they have compared to things farther apart [54]. In this sense, the IDW will use the measured values to predict a value for any surrounding unmeasured location. These measured values closest to the prediction location will have more influence from those farther away. Thus, IDW assumes that each measured point has a location influence that diminishes with distance.

The IDW spatialization was performed using training samples defined in the ML methodology (*section* 2.4). Therefore, this step comprehends the characteristics of only evaluate the performance of spatialization. For the IDW, at least five sampled points closer to the pixel were used to spatialize the soil attributes evaluated (clay, sand, CEC and P). The spatialization was performed using the *krige* function of the *gstat* package in *R* software [40,55].

In OK, we performed the semi-variogram analysis to evaluate the spatial dependence of soil attributes using the *gstat* [55] package in *R* software [40]. Spatial dependence is the statistical relationship between the values of the variates measured in samples and the geographical position of these samples. A semi-variogram is an analysis that demonstrate spatial dependence of variates through the relationship between the semi variance of the values and the distance between all sample points.

Firstly, we analyzed the total dataset and three semi-variogram models were performed for all datasets: Spherical, Exponential and Gaussian. At this stage, it was possible to verify that for all soil attributes, the Exponential model demonstrated the most efficiency and, therefore it was chosen automatically by *autofitvariogram* in *automap* [56] package [40]. This information was used to determine the semi-variogram of the training samples for the next phase of the spatialization of soil attributes. The phenomenon of the pure nugget effect was also evaluated. It would not be specialized if all the tested models obtained this nugget effect phenomenon for the soil attributes. Secondly, we performed separation of the total dataset in the same manner we performed IDW.

An automatic semi-variogram calculation function follows the assumptions of less human intervention in spatializing soil attributes by the OK method. The spatialization by kriging was performed using at least five points closest to pixel to be specialized which was performed using the kriging function of the *gstat* package [55].

The 100 maps were generated for each attribute (clay, sand, CEC and P), to IDW and OK, being used in the final maps composed by the average value and correlation coefficient values (CV %) of the 100 maps for each pixel.

### Performance of spatialization methods

To evaluate the model’s and spatialization performance, we applied the fitted model to test data and the accuracy was expressed by the following statistical indexes: R-squared (R^2^) (Eq. (1)), root mean squared error (RMSE) (Eq. (2)) and, mean absolute error (MAE) (Eq. (3)).


$$\begin{array}{c} R^{\text{2}}=\frac{\sum_{i=\text{1}}^{n}{\left(P_{i}-\underset{―}{Om}\right)}^{\text{2}}}{\sum_{i=\text{1}}^{n}{\left({Om}_{i}-\underset{―}{Om}\right)}^{\text{2}}} \end{array}$$ (1)

$$\begin{array}{c} RMSE={[\frac{\text{1}}{N}\sum_{i=\text{1}}^{N}{\left(P_{i}-O_{i}\right)}^{\text{2}}]}^{\frac{\text{1}}{\text{2}}} \end{array}$$ (2)

$$\begin{array}{c} MAE=\;\frac{\sum_{i=\text{1}}^{N}\left|P_{i}-O_{i}\right|}{n} \end{array}$$ (3)

The results of R², RMSE and MAE from the 100 rounds of the tested spatialization methods (ML, OK and IDW) were to calculate the final result. Those summarized by the mean and standard deviation of R², RMSE and MAE.

For comparison purposes, with results generated for each element, the RMSE and MAE were calculated for the null model (NULL_RMSE and NULL_MAE). The null model is a simple model which work as a basis for comparison with other models and that adjusts a simple (mean) function as a predictor. The null model considers using the average value of all data of the properties of the soil (Eq. (4) and Eq. (5)); the information that best represents these data is the average. Therefore, any method that presents RMSE and MAE values greater than those found by the NULL models must be discarded. The RMSE_NULL and MAE_NULL were obtained by the *care*t package’s nullMode function [45].


$$\begin{array}{c} NULL\_RMSE={[\frac{\text{1}}{N}\sum_{i=\text{1}}^{N}{\left(\underset{―}{{Om}_{t}}-O_{i}\right)}^{\text{2}}]}^{\frac{\text{1}}{\text{2}}} \end{array}$$ (4)

$$\begin{array}{c} NULL\_MAE=\frac{\sum_{i=\text{1}}^{N}\left|{Om}_{t}-O_{i}\right|}{n} \end{array}$$ (5)

The non-parametric Kruskal–Wallis’s test at 5% significance, with the accuracy parameter being evaluated. Dunn’s post-hoc test of multiple comparisons [57] were performed for the accuracy values that presented statistically significant differences between the algorithms.

### Computational processing time of spatialization methods

The processing time was measured by the difference between the start and end times to evaluate the processing time per run. The initial time was considered corresponding to data entry in the processing cycle, while the final time was considered corresponding to map generations. For that, we used the *proc.time* function of *R* software [40].

All processing was run on the same computer: Dell Precision Tower 3620 model; Intel® Xeon® processor E3-1270 v5 with 4 cores and 8 treads; 32GB of RAM DDR4 2133 Mhz; Nvidea® Quadro 620 graphics card; storage HD data 480GB Kingston A400 SSD and Windows 10® pro–operating system. Computer parallelism was used for data processing where the number of treads was 7 of the 8 available treads. This choice was made so that the processor would work with slack so that the processor would not overheat and, consequently, the *throttling* process, which can negatively affect the processing time of the rounds.

### Spatial autocorrelation analysis (Moran’s I)

Spatial autocorrelation of the soil attributes (sand, clay, P, and CEC) was assessed using the global Moran’s I index. The analysis was performed in R software [58], based on the geographic coordinates of all sampling points and a standardized Euclidean distance matrix. Statistical significance was determined using the analytical expectation of Moran’s I under the null hypothesis of spatial randomness. Positive and significant Moran’s I values denote the presence of spatial structure, whereas non-significant values indicate a random spatial pattern.

### Data and materials

The data and scripts used in this processing are available on Zenodo under the https://doi.org/10.5281/zenodo.18166627.

## Results

### Models’ performance and uncertainty

In general, the worst performance in modeling soil attributes occurred for IDW and OK (Table 1). The ascending order of the R^2^ values and, consequently the prediction performance for the test dataset were: 0.526, 0.580, 0.665 and 0.669 for CEC, P, clay and sand, respectively by OK. All the values are similar to those obtained by IDW, differing only for sand and clay content (R^2^ = 0.653 and 0.675, respectively). In addition, Table 2 presents the statistical differences in R^2^ values (determined using the Kruskal-Walli’s test) when comparing traditional geostatistical methods with ML algorithms, highlighting the various performance outcomes. It can be observed that the Random Forest (RF) machine learning algorithm demonstrated superior performance, with statistically significant differences compared to traditional geostatistical methods, across all evaluated soil attributes, except for CEC (Table 2).

**Table 1:** Models’ performance for Inverse Distance Weight (IDW) and Ordinary Kriging (OD) for all soil attributes, based on R^2^, RMSE, MAE and RMSE_NULL/MAE_NULL.

Performance	Soil Attributes		IDW	OK	RMSE_NULL/MAE_NULL
R²	Sand	Train	0.675	0.669	–
		Test	0.675	0.669	
	Clay	Train	0.653	0.665	–
		Test	0.653	0.665	
	P	Train	0.586	0.580	–
		Test	0.586	0.580	
	CEC	Train	0.523	0.526	–
		Test	0.523	0.526	
RMSE	Sand	Train	10.12	10.18	17.65
		Test	10.12	10.23	
	Clay	Train	6.78	6.77	11.50
		Test	6.78	6.64	
	P	Train	21.54	19.28	31.50
		Test	21.54	20.56	
	CEC	Train	3.55	3.45	4.96
		Test	3.55	3.45	
MAE	Sand	Train	6.23	6.23	14.05
		Test	6.00	6.02	
	Clay	Train	4.48	4.48	9.36
		Test	4.38	4.35	
	P	Train	7.50	6.84	17.54
		Test	7.50	7.41	
	CEC	Train	2.42	2.42	3.53
		Test	2.34	2.34	

**Table 2:** Model performance was systematically evaluated using distinct grid-based spatial covariate designs: a 2 × 2 moving window including the central pixel and X–Y spatial coordinates (7 covariates); a 5 × 5 window (27 covariates); a 7 × 7 window (51 covariates); and a 10 × 10 window (102 covariates), implemented across four machine learning algorithms.

Clay		Sand		Cation Exchange Capacity		Phosphorus	
R^2^	Grid + Interpolator	R^2^	Grid + Interpolator	R^2^	Grid + Interpolator	R^2^	Grid + Interpolator
0.68 a	X, Y 10x10 RF	0.70 a	X, Y 5x5 RF	0.53 a	OK	0.62 a	X, Y 2x2 center RF
0.68 b	X, Y 7x7 RF	0.70 b	X, Y 10x10 RF	0.52 ab	IDW	0.62 a	X, Y 5x5 RF
0.67 bc	X, Y 5x5 RF	0.70 b	X, Y 7x7 RF	0.51 b	X, Y 7x7 RF	0.61 abc	X, Y 7x7 RF
0.67 bcd	X, Y 2x2 center RF	0.69 bc	X, Y 2x2 center SVM	0.51 bc	X, Y 5x5 RF	0.60 ab	X, Y 10x10 RF
0.66 bcd	OK	0.69 bc	X, Y 7x7 SVM	0.51 bcd	X, Y 10x10 RF	0.60 abcd	X, Y RF
0.65 cde	IDW	0.69 bcd	X, Y 10x10 SVM	0.50 cd	X, Y 2x2 center RF	0.60 abcd	X, Y Cubist
0.65 cdef	X, Y 2x2 center Cubist	0.69 bcdef	X, Y 5x5 SVM	0.50 cd	X, Y RF	0.59 abcd	X, Y 2x2 center Cubist
0.64 cdef	X, Y 2x2 center SVM	0.69 bcde	X, Y 2x2 center RF	0.46 cde	X, Y 5x5 Cubist	0.59 abcd	IDW
0.64 defg	X, Y 5x5 SVM	0.68 bcdef	X, Y SVM	0.46 cde	X, Y 2x2 center Cubist	0.59 abcd	X, Y 5x5 Cubist
0.64 efgh	X, Y RF	0.68 cdef	IDW	0.46 cde	X, Y 10x10 Cubist	0.58 abcd	OK
0.64 efgh	X, Y 10x10 SVM	0.68 cdef	X, Y 2x2 center Cubist	0.46 de	X, Y 2x2 center SVM	0.57 bcd	X, Y SVM
0.64 efgh	X, Y 7x7 SVM	0.67 cdef	X, Y Cubist	0.46 de	X, Y 5x5 SVM	0.57 bcd	X, Y 7x7 Cubist
0.64 efgh	X, Y Cubist	0.67 cdef	OK	0.46 def	X, Y SVM	0.56 cd	X, Y 10x10 Cubist
0.64 efgh	X, Y 5x5 Cubist	0.66 def	X, Y 5x5 Cubist	0.45 de	X, Y Cubist	0.55 d	X, Y 7x7 SVM
0.63 efgh	X, Y 7x7 Cubist	0.66 ef	X, Y RF	0.45 defg	X, Y 7x7 SVM	0.55 d	X, Y 10x10 SVM
0.63 efgh	X, Y SVM	0.66 ef	X, Y 10x10 Cubist	0.45 efgh	X, Y 10x10 SVM	0.55 d	X, Y 5x5 SVM
0.62 efgh	X, Y 10x10 Cubist	0.64 ef	X, Y 7x7 Cubist	0.45 efgh	X, Y 7x7 Cubist	0.55 d	X, Y 2x2 center SVM
0.60 efgh	X, Y 7x7 EARTH	0.64 ef	X, Y 2x2 center EARTH	0.43 gh	X, Y 10x10 EARTH	0.54 d	X, Y 2x2 center EARTH
0.60 fgh	X, Y 2x2 center EARTH	0.63 ef	X, Y 7x7 EARTH	0.40 fgh	X, Y 7x7 EARTH	0.48 d	X, Y EARTH
0.60 gh	X, Y 10x10 EARTH	0.62 ef	X, Y 10x10 EARTH	0.40 gh	X, Y 5x5 EARTH	0.48 d	X, Y 10x10 EARTH
0.57 h	X, Y 5x5 EARTH	0.62 ef	X, Y 5x5 EARTH	0.39 h	X, Y 2x2 center EARTH	0.47 d	X, Y 5x5 EARTH
0.41 h	X, Y EARTH	0.55 f	X, Y EARTH	0.33 h	X, Y EARTH	0.47 d	X, Y 7x7 EARTH

The results demonstrated clear performance distinctions among the baseline methods (OK, IDW, and standalone ML models) and the proposed Euclidean-grid ML framework. Baseline geostatistical approaches (OK and IDW) showed consistently lower predictive accuracy, while standalone ML models using only X–Y coordinates also underperformed relative to grid-enhanced ML models across all evaluated soil attributes. These baseline comparisons establish a consistent benchmark from which the added benefits of the Euclidean-grid integration can be quantified.

**Fig 4:**
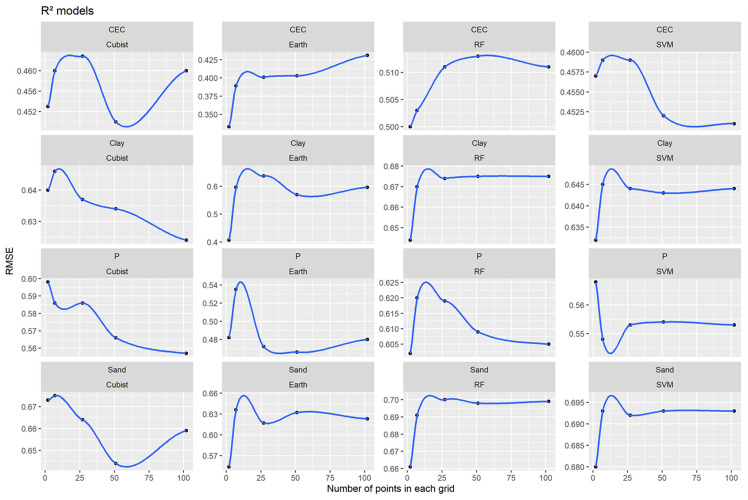
Models’ performance for Machine Learning algorithms and different grids systems for all soil attributes based on R^2^.

Among the worst performance for ML algorithms the ascending order of the R^2^ values was: 0.333, 0.407, 0.466 and 0.554 for CEC, clay content, P and sand content, respectively by Earth algorithms (Fig 4). The worst grids were X, Y for all soil attributes, except for the P whose worst grid was 7 x 7 + X, Y (Fig 4). On the other hand, the best model’s performance was obtained by RF. The increasing order of the R^2^ values and consequently, the model’s performance were: 0.513, 0.620, 0.675 and 0.700 for CEC, P, clay and sand content, respectively (Fig 4). The best grids varied among soil attributes. In general, the best grids were between 2 x 2 + X, Y + central and 10 x 10 + X, Y (Fig 4

The worst results of RMSE and MAE were achieved by IDW and OK for the soil attributes sand, clay and P (Table 1). Thus, for CEC it presented the best result, with the lowest RMSE and MAE values. The IDW obtained the best result for sand content and CEC, with the lowest MAE values, where for CEC these values were similar to those obtained for OK (Table 1).

**Fig 5:**
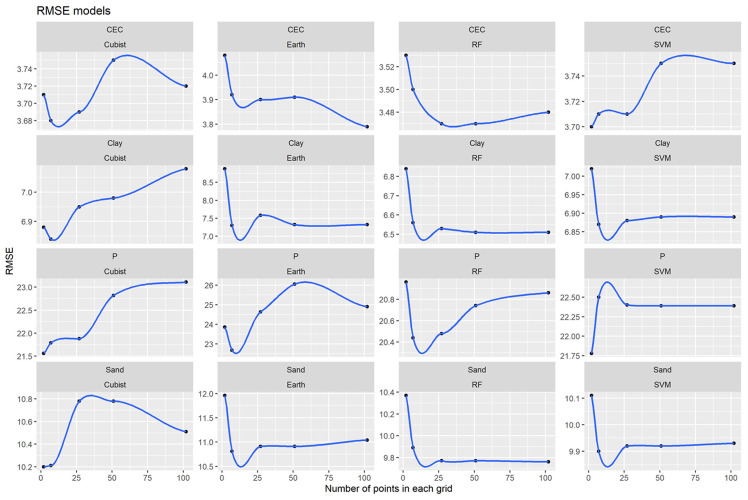
Models’ performance for Machine Learning algorithms and different grids systems for all soil attributes based on RMSE.

**Fig 6:**
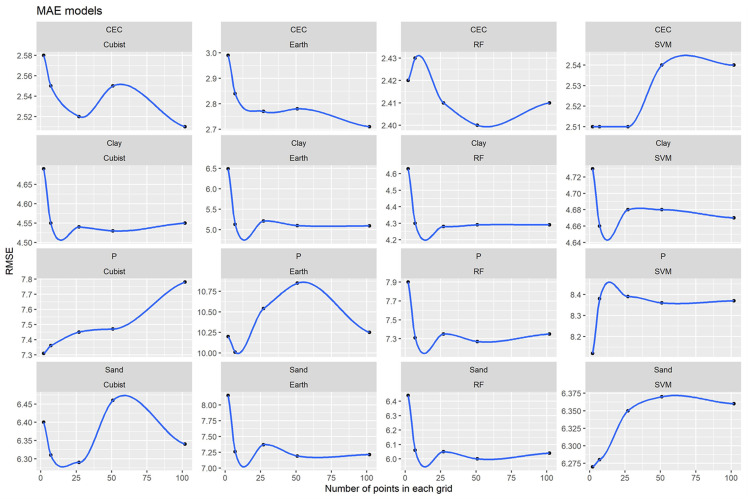
Models’ performance for Machine Learning algorithms and different grids systems for all soil attributes based on MA.

In general, the best RMSE and MAE results were obtained for the ML algorithms (Table 1, Fig 5 and 6). However, this trend only did not occur for CEC and sand (MAE).

The assessment of spatial dependence using Moran’s I (Table 3) revealed significant positive autocorrelation for all soil attributes evaluated. This outcome is consistent with the expected behavior of pedological variables, which are typically structured by continuous environmental and pedogenetic processes across the landscape. Observed Moran’s I values were moderate for texture-related variables (sand: *I* = 0.17; clay: *I* = 0.21) and weaker but still significant for CEC (*I* = 0.13; *p* < 0.001). Phosphorus exhibited the strongest spatial dependence (*I* = 1.69; *p* < 0.001), suggesting a highly clustered distribution likely influenced by local soil–plant interactions or management effects. Overall, the highly significant *p*-values (all *p* < 0.001) indicate that the spatial patterns in the dataset are not random, confirming the presence of structured spatial dependence across the study area.

**Table 3:** Global Moran’s I values (observed statistic and *p*-value) for the analyzed soil attributes.

Soil Attributes	Observed Moran I	P valor
Sand	0.170	1.390e-08
Clay	0.210	3.920e-12
Phosphorus	1.700	0.000e + 00
Cation Exchange Capacity	0.130	1.064e-05

### Spatialization of soil attributes by the predicted models and processing time

**Fig 7:**
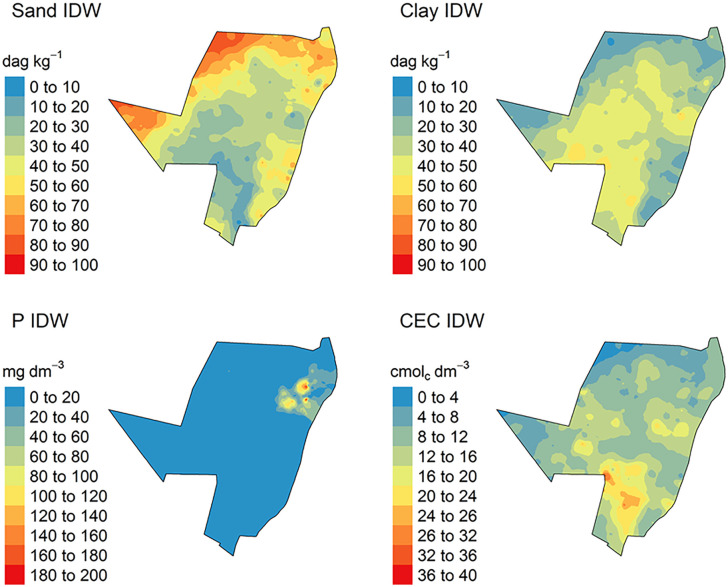
Spatialized soil attribute data by Inverse Distance Weighted (IDW) method. P: phosphorus content; CEC: cation exchange capacity.

**Fig 8:**
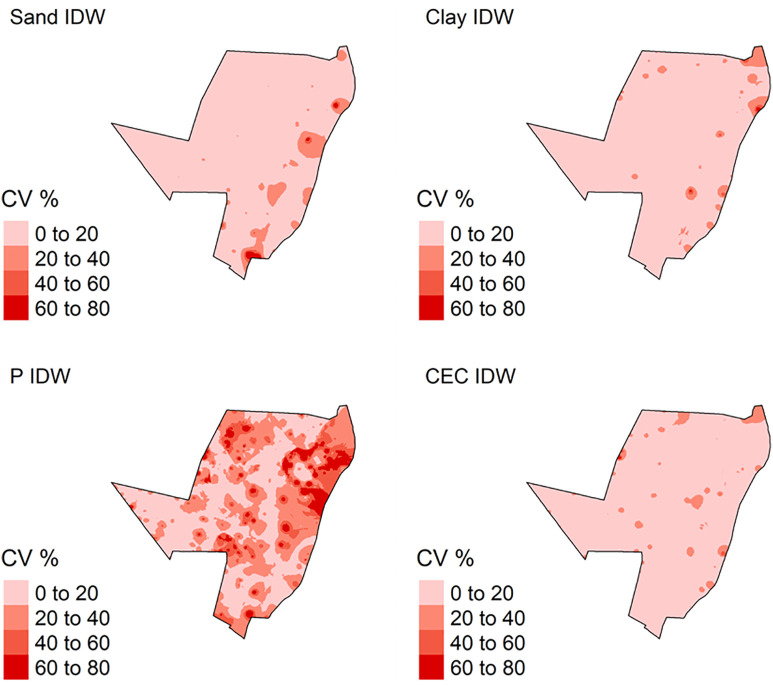
Coefficient of variation (CV) for soil attributes by Inverse Distance Weighted (IDW) method. P: phosphorus content; CEC: cation exchange capacity.

**Fig 9:**
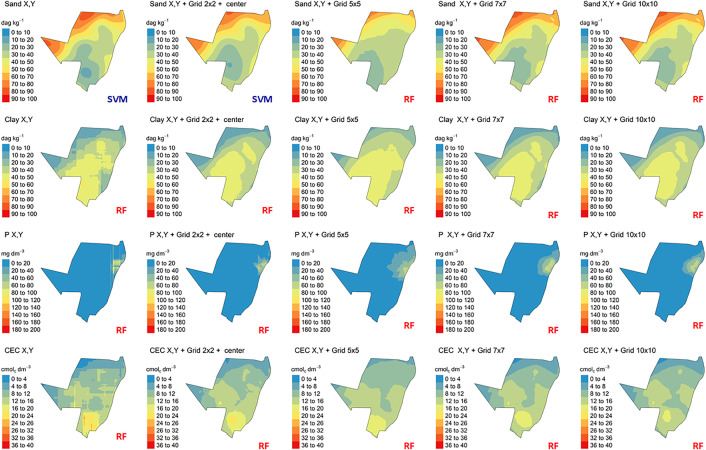
Spatialized soil attribute data by Machine Learning methods (ML): P: phosphorus content; CEC: cation exchange capacity; Sand content X, Y predicted and spatialized by Support Vector Machine (SVM); Sand content X, Y + grid 2 x 2 + center predicted by Support Vector Machine (SVM); all others soil attributes and grid systems predicted and spatialized by Random Forest (RF).

The spatial variations from the predicted models for CEC, P sand and clay content by IDW are demonstrated in Fig 7, while for OK in Fig 8. For ML methods, which used different grid systems, the spatial variation from the predicted models is illustrated in Fig 9.

**Fig 10:**
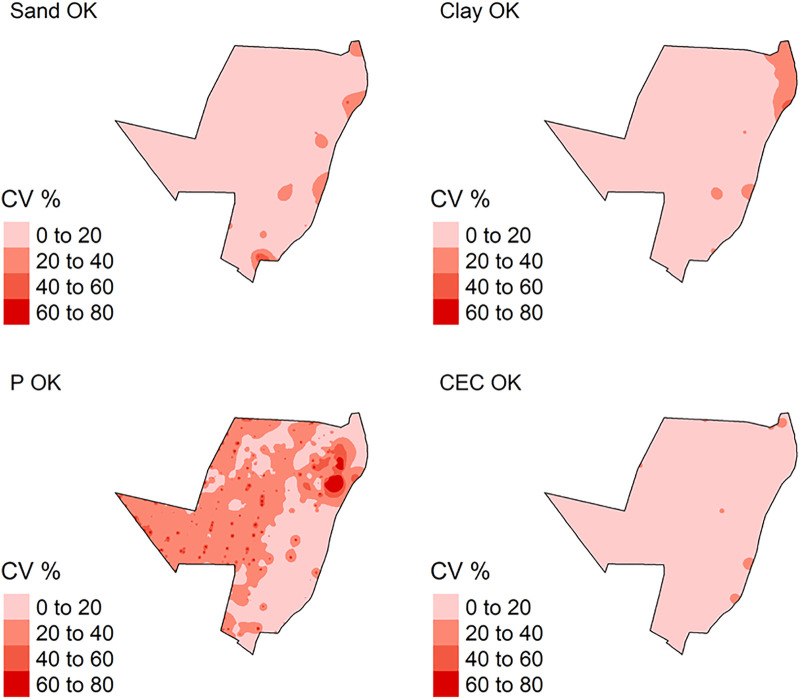
Coefficient of variation (CV) for soil attributes by Ordinary Kriging (OK) method. P: phosphorus content; CEC: cation exchange capacity.

The total sand and clay content predicted by IDW varied from 0 to 100 dag k^-1^, (Fig 7). In addition, P and CEC varied from 0 to 200 mg dm^-3^ and 0–40 cmol_c_ dm^-3^, respectively (Fig 7). The analysis of the uncertainty of prediction (coefficient of variation) varied from 0 to 80%, for all soil attributed modelled (Fig 10).

For OK, the total sand and clay content predicted varied from 0 to 100 dag k^-1^. P and CEC varied from 0 to 200 mg dm^-3^ and 0–40 cmol_c_ dm^-3^, respectively (Fig 8). The analysis of the uncertainty of prediction (coefficient of variation) varied from 0 to 60% for sand and clay content, and P and CEC (Fig 9).

**Fig 11:**
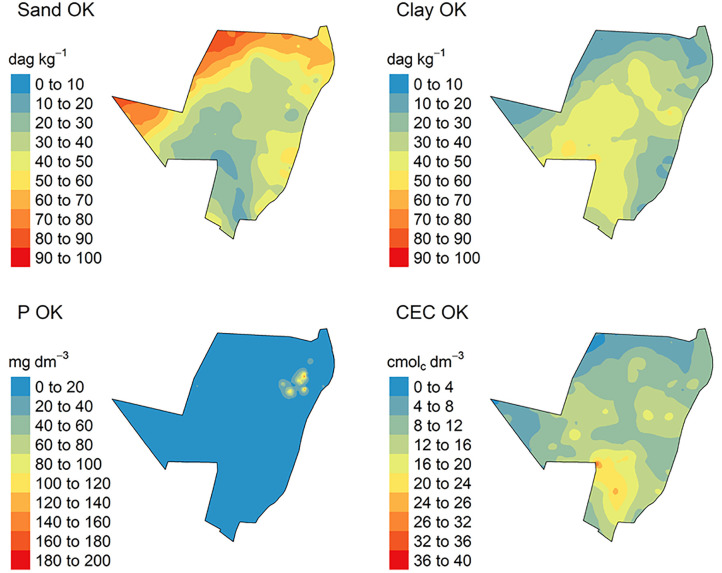
Spatialized soil attribute data by Ordinary Kriging (OK) method. P: phosphorus content; CEC: cation exchange capacity.

The predicted model varied among the different ML algorithms tested (Fig 11). However, in general, the RF presented the best results for all soil attributes, except for sand content in X, Y and X, Y + grid 2 x 2 + center grid system, which the best results were obtained by SVM (Fig 11).

**Fig 12:**
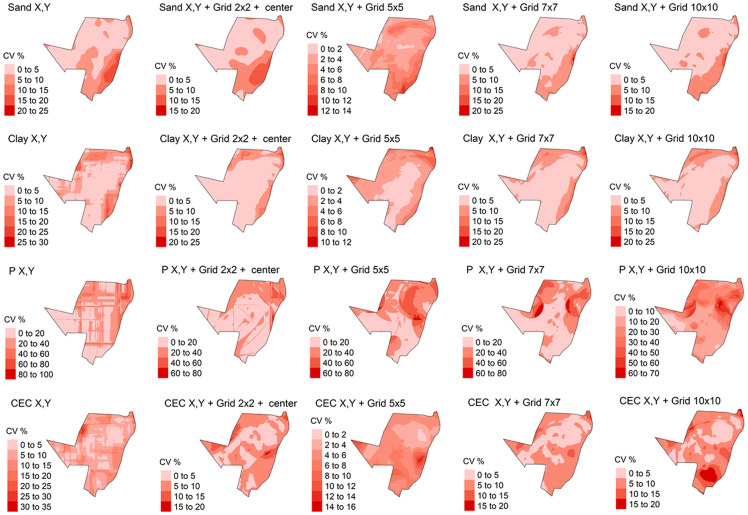
Coefficient of variation (CV) for soil attributes by Machine Learning methods (ML): P: phosphorus content; CEC: cation exchange capacity; Sand content X, Y predicted and spatialized by Support Vector Machine (SVM); Sand content X, Y + grid 2 x 2 + center predicted by Support Vector Machine (SVM); all others soil attributes and grid systems predicted and spatialized by Random Forest (RF).

For soil physical attributes, the total sand and clay content predicted by the RF algorithm varied from 0 to 100 dag k^-1^ by X, Y + 5 x 5 grid system (Fig 11). The sand and clay content predicted by the RF algorithm varied from 0 to 100 dag k^-1^ by the Y, Y + 7 x 7 grid system and 10 x 10 + X, Y grids, which presented the same values and presented the bests performance (Fig 5 and 11). The analysis of the uncertainty of prediction (coefficient of variation), in general varied from 0 to 25%, for sand and 0–30% for clay content (Fig 12). For some soil attributes, the map presented predictions in lines with abrupt transitions for some maps regions, clearly visible in clay content, P and CEC in X, Y grid system by RF (Fig 11).

For soil chemical attributes, the P spatial variation by the RF algorithm is shown in Fig 11. The P content predicted ranged from 0 to 200 mg dm^-3^ (Fig 11), while the analysis of the uncertain of the prediction varied from 0 to 100% among different grid systems (Fig 12). The spatial variations from the predicted models for CEC by RF are demonstrated in Fig 6. The CEC predicted varied from 0 to 40 cmol_c_ kg^-1^ (Fig 11). In addition, the coefficient of variation varied from 0 to 35% for CEC, among different grids systems (Fig 12).

**Fig 13:**
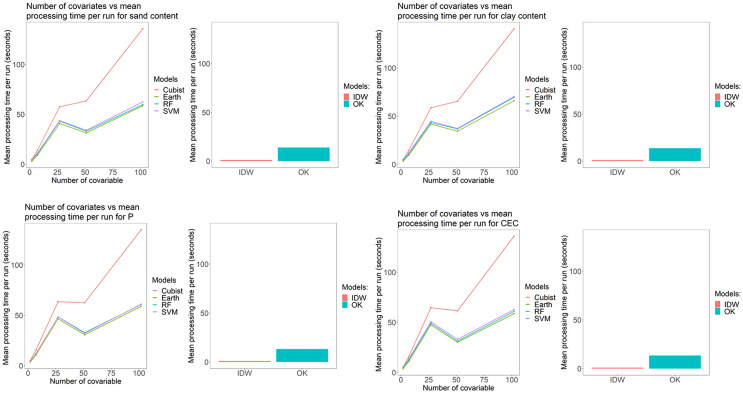
Mean processing time per run *vs* number of covariates for each soil attribute and each method tested: P: phosphorus content; CEC: cation exchange capacity; Multivariate Adaptive Regression Spline (Earth); Random Forest (RF); Support Vector Machine (SVM); Inverse Distance Weighted (IDW); Ordinary Kriging (OK).

The time spent in computer processing, per training/spatialization cycles *versus* the number of covariates is shown in Fig 13, for all spatialization methods used (IDW, OK and ML). The IDW presented the shortest time interval for processing, ranging from 2 to 10 seconds (Fig 13). OK presented the second minor time to total processing 13 approximately seconds (Fig 13). Machine learning algorithms presented varying time intervals to computer processing, according to the algorithms used. Earth had the shortest time interval, followed by RF, SVM and Cubist, respectively (Fig 13). For all algorithms tested the grids of 7 x 7 + X, Y obtained less time per processing cycle, when compared to the smaller set of 5 x 5 + X, Y covariates, excepted for Cubist algorithms (Fig 13).

**Fig 14:**
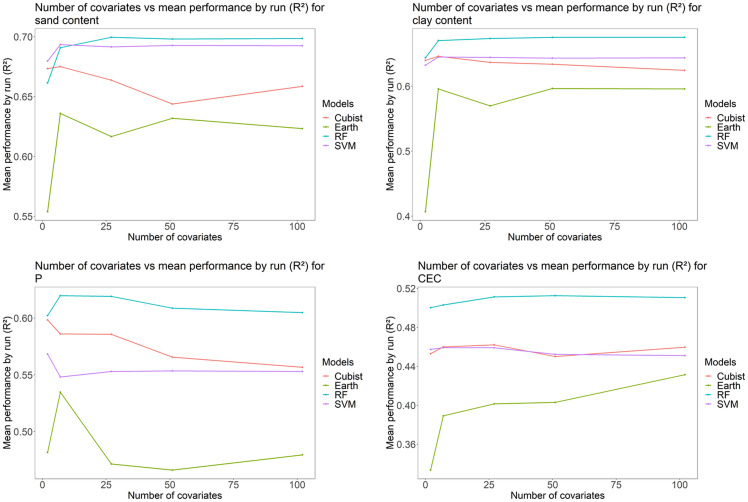
Mean performance (R^2^) by run *vs* number of covariates for each soil attribute and each method tested: P: phosphorus content; CEC: cation exchange capacity; Multivariate Adaptive Regression Spline (Earth); Random Forest (RF); Support Vector Machine (SVM); Inverse Distance Weighted (IDW); Ordinary Kriging (OK).

The relationship between the mean performance of models (R^2^) by run and numbers of covariates existing for each grid system used in ML methods are shown in Fig 14. Earth had the shortest time interval, followed by Cubist, SVM and RF, respectively (Fig 14).

## Discussion

### Model’s performance and uncertainty

The superior performance of ML compared to traditional geostatistical methods for clay, sand, and P may be attributed to a specific dependency associated with geostatistical methods, which is not necessary for ML [59]. OK and IDW methods have the worst performance when compared to ML algorithms. This was expected because OK and IDW use Euclidean distance and/or semi- variogram as a basis, in addition to the punctual values for the analyzed attributes [60,61]. On the other hand, ML algorithms can use a greater number of covariates to predict attributes [24,41], which in this case were our grid and/or coordinate systems. Similar results were obtained by Fathololoumi [25], when comparing OK, IDW and ML for mapping the local variation of soil moisture.

Our findings align with an emerging body of evidence demonstrating the superior predictive capacity of machine learning approaches in digital soil mapping. Studies such as Hengl et al. (2017) [27], Behrens et al. (2018) [26], and Viscarra Rossel et al. (2014) [62] similarly report that algorithmic flexibility and the ability to incorporate numerous spatial covariates allow ML models to outperform traditional geostatistical methods. The consistent advantage of Random Forest observed in our results mirrors the conclusions of Gomes et al. (2019) [42] and Rastgou et al. (2020) [63], who also identified RF as the most robust algorithm for handling nonlinear soil-landscape relationships. Our comparative evaluation reinforces this trend by showing that ML models enhanced with Euclidean-grid covariates achieve accuracy levels unattainable by OK and IDW, which rely on stronger assumptions regarding spatial dependence.

The comparative evaluation against baseline methods confirms that the performance improvements achieved by our framework are not merely algorithmic, but stem directly from the incorporation of Euclidean-distance-based spatial covariates. While OK and IDW rely on inherent spatial autocorrelation assumptions, and standalone ML models operate without explicit spatial structure, the Euclidean-grid approach provides richer spatial information, enhancing model generalization and reducing prediction error. This benchmarking demonstrates that the proposed framework extends beyond traditional interpolation and standard ML practices, offering a demonstrable methodological advance.

The Earth presented the worst performance for CEC, clay content, P and sand content (Fig 4). This algorithm creates linear regressions on defined ranges of values relative to each evaluated attribute (*spline*), adjusting these values within the established range [53,64]. However, some of the predicted and specialized soil attributes may not present a linear relationship with covariates. Considering the grids systems, the worst performance was X, Y for all soil attributes (excepted for P) (Fig 4). This was expected due to the small number of covariates used for prediction. This result is corroborated by [26] who found inferior performances when they used fewer covariates when comparing two grids systems when they compared two grids systems used a small number of covariates.

The thematic structure of prediction errors and uncertainty patterns observed here highlights the interaction between spatial processes and algorithm behavior. The weaker performance of Earth and standalone X-Y models suggests that certain soil attributes, such as phosphorus or cation exchange capacity, are governed by complex spatial gradients that cannot be captured without explicit spatial descriptors. The grid-based Euclidean covariates act as surrogate spatial predictors, allowing the ML algorithms to reconstruct spatial structure indirectly, thereby overcoming one of the major limitations in purely attribute-driven ML approaches.

RF obtained the best model’s performance for all soil attributes (Fig 4). This can probably be explained because the RF is a more generalist model capable of operating in various conditions of sample distributions and input covariates [65–67] and, therefore this algorithm is more sensitive [68]. In addition, the RMSE referring to the RF algorithm presented a defined trend for all the evaluated soil attributes (Fig 5). The general behavior of the curves decreases for all attributes and tended to stabilize with an increase in the number of grids (covariates). For the Cubist algorithm, the opposite occurred, which there was a general tendency to increase the RMSE values. This indicates that algorithms that use only one tree may not be suitable for use with spatial data (grids). On the other hand, algorithms that use multiple trees such as RF can achieve better performance when operates with spatial data. Also, the RF requires no external testing as it performs its internal validation using methods such as *bootstrap* [69]. RF is one of the best-performing algorithms for predicting various soil attributes [63,70–73]. In general, the best grids varied among soil attributes (Fig 4). This soil variability can be attributed to the heterogeneity of spatial distribution of soil attributes [74]. For example, clay and sand content presented a more homogeneous distribution, resulting in fewer grids.

There is a tendency for the best R^2^ (higher values), RMSE and MAE (lower values) results to be equivalent for all attributes (Fig 4, 5 and 6). However, for sand content, MAE values did not follow this trend. This occurred because the MAE has the least susceptibility to extreme values [75], which was impossible to evaluate in this research.

The lower MAE and RMSE values found in the best results for the predicted (soil attributes) when compared to the values of NULL_RMSE and NULL_MAE show that IDW, OK and ML models have smaller errors than the use of mean values for the entire area (Table 1, Fig 5 and 6) and therefore, present better performance and accuracy.

A key strength of the proposed framework lies in its scalability and independence from environmental covariates, enabling spatial prediction even in data-poor regions where remote sensing or environmental layers are unavailable. However, the approach also has limitations: (i) performance depends on an adequate representation of the spatial domain by the grid structures; (ii) computational cost increases with grid size; and (iii) the framework does not explicitly model spatial autocorrelation, which may limit interpretability compared to geostatistical approaches. These trade-offs must be considered when selecting the most appropriate method for operational soil mapping tasks.

The substantially higher uncertainty observed for P predictions (CV up to 100%) can be explained by the inherently patchy spatial behavior of soil phosphorus. Unlike texture or CEC, which reflect parent material and pedogenetic processes acting at broader spatial scales, available P is strongly influenced by highly localized biogeochemical dynamics [76], such as litter deposition, root uptake, organic matter hotspots, and mineralogical reactions affecting adsorption and desorption. These processes create sharp micro-scale gradients that are difficult to capture using spatial covariates based solely on Euclidean distance. Additionally, the P dataset exhibited extreme values and high skewness, which tend to inflate uncertainty in both geostatistical and machine-learning predictions [76]. Consequently, even high-performing ML models show greater variability in P estimates across resampling iterations, reflecting the genuine fine-scale heterogeneity of this attribute.

The presence of significant spatial autocorrelation across all soil attributes demonstrates that the dataset contains sufficient spatial structure to justify the application of spatial interpolation methods [77], particularly geostatistical approaches such as ordinary kriging, which rely directly on the continuity of spatial processes to generate accurate predictions. The structured spatial patterns identified here are consistent with the pedogenetic controls that shape soil variability at landscape scales, including topography-driven redistribution, parent-material contrasts, and biogeochemical gradients.

At the same time, the detected spatial dependence is equally informative for ML models. Although ML algorithms do not explicitly require spatial continuity, their predictive performance can benefit substantially from the presence of spatial gradients and environmental clustering. Such structure enhances the model’s ability to learn spatially coherent relationships between soil properties and the covariates used as predictors [78]. Therefore, the identification of spatial dependence in the dataset strengthens the methodological framework of this study. It confirms that the data contain meaningful spatial signals that can be leveraged by both geostatistical techniques and machine-learning approaches. Importantly, the observed autocorrelation does not represent a methodological issue; instead, it provides empirical support that spatial information is embedded in the soil attributes, enabling more reliable and interpretable predictions across the study area.

The presence of significant spatial autocorrelation demonstrates that the dataset contains sufficient spatial structure to justify the use of geostatistical methods, such as kriging, which depend on spatial continuity to generate unbiased and minimum-variance estimates [79,80]. This structured spatial signal is equally relevant for machine-learning algorithms. Although ML models do not rely on explicit probabilistic assumptions about spatial dependence, their predictive performance can benefit from underlying environmental gradients and localized neighborhood structure, which help stabilize learned relationships and improve generalization in spatial domains [81,82]. Detecting spatial dependence therefore supports the complementary use of geostatistical and machine-learning approaches, since both classes of methods can exploit meaningful spatial patterns embedded in the data. In this context, spatial autocorrelation is not a methodological limitation but rather a confirmation that the dataset contains informative spatial signals that can be leveraged by different predictive frameworks to produce more reliable soil attribute estimates [83,84]

### Predicted variates spatialization and processing time

All specialized maps have the same resolution and sampling arrangement (Fig 7, 8, 9). IDW and OK had a larger sampling range for all soil attributes, in other words, the major difference between the highest and lowest values for predicted soil attributes (Fig 7 and 8). This is related to the spatial dependence of the data, which is important for predicting attributes by OK and IDW methods [85,86]. Furthermore, IDW and OK can be more influenced by extreme values of predicted variables [87,88]. As result, these values influence the prediction of soil attributes in the neighborhood areas. In addition, this effect produced “bulls-eye”, for example P (Fig 7 and 8) when there are extreme values samples concentrated in some area [89,90]. The maps spatialized by IDW (Fig 7) are visually more heterogeneous and provide greater detail, when compared to maps generated by OK (Fig 8). These differences are due to the functions used by each model to spatialize soil attributes. IDW uses the inverse squared sample distance [60], while OK calibrates a spatial variability function across all samples (semi-variogram) [91]. As the semi-variogram uses all points, there is a reduction in the degree of detail [92].

The maps generated by ML showed smaller areas with extreme values compared to IDW and KO (Fig 9). Machine learning algorithms use point samples to create models, which are used to predict the contents of soil attributes [42]. These algorithms create linear or non-linear functions that do not depend on samples’ values anymore to predict the attributes [93,94]. Therefore, these models are less influenced by extreme values of attributes.

The internal characteristics of each ML algorithm affected how the maps of soil attributes were spatialized (Fig 9). In general, the best specialization for all soil attributes was performed by the RF algorithm, except for grid systems 2 x 2, X, Y and X, Y (Fig 9). Evaluating the spatialization results by ML algorithms, it was observed that the maps are similar in relation to the estimated value for all grid systems (Fig 9), considering the best prediction results for the spatialized attributes. However, the characteristics and numbers of samples of each spatialized variable influenced how each model operated during the spatialization and, therefore, the differences between the maps generated for each attribute, by each algorithm and by each grid system [41].

The IDW presented the lowest mean processing time per run cycle, about all other tested methods (Fig 13). The explanation is related to the lower spatialization complexity where only one function is applied, which considers only a base function related to the distance of the sampling points [60,95].

Ordinary kriging presented the second-lowest mean processing time (Fig 13). This is explained because this method needs to create a semi-variogram which is a longer process than the equation used by the IDW [96]. Regarding ML algorithms, the processing time was longer compared to OK, since the training and testing steps require more input information in the models for spatialization.

With increasing covariates, the difference in processing time increases linearly (Fig 13). Among the algorithms tested, the Cubist showed the highest processing time per run (Fig 13). The difference in processing time between Cubist and the other algorithms was notably perceived from the set of 7 covariates (Grid 2 x 2 + center + X, Y). This is related to Cubist’s internal characteristics when spatializing, which may be associated with how the algorithm separates covariates [97,98]. The RF, SVM and earth algorithms, made it impossible to notice significant differences in time per processing cycle, within the same set of covariates (Fig 13). This may be related to the fact that these algorithms have already achieved a satisfactory optimization to be used in R software. Also, these small differences can be associated with internal computer limitations.

A clear trade-off emerged between predictive accuracy and computational efficiency across the evaluated methods (Table 4). IDW was consistently the fastest technique, requiring only simple distance-based calculations, but it also yielded the lowest predictive accuracy. Ordinary Kriging presented slightly higher computational cost due to variogram modeling, yet its accuracy remained comparable to IDW. Among machine learning algorithms, Earth exhibited the shortest processing time but also the weakest predictive skill, reflecting its limited ability to capture nonlinear spatial patterns. Cubist, SVM, and Random Forest showed progressively higher computational demands, especially under larger grid systems, but also delivered substantial gains in predictive performance. Notably, Random Forest achieved the highest accuracy for all soil attributes, albeit at the expense of markedly increased processing time. These results demonstrate that improvements in spatial prediction come with higher computational cost, and that optimal method selection depends on the balance between performance objectives and available computational resources.

**Table 4:** Summary of predictive accuracy and computational cost for spatialization methods.

Method	Predictive Accuracy	Computational Cost	Strengths/ Notes
IDW	Low	Very Low (Fastest)	Simple; sensitive to extreme values; no uncertainty estimation
Ordinary Kriging (OK)	Low–Moderate	Low–Moderate	Variogram-based; smoother predictions; needs spatial structure
Earth (MARS)	Low	Very Low	Fast ML; limited for nonlinear spatial patterns
Cubist	Moderate	High	Hybrid tree-regression; cost increases with many covariates
SVM (Radial)	High	High	Captures nonlinear relationships; computationally intensive
Random Forest	Highest	High–Very High	Most robust; accuracy increases with grids but higher cost

RF presented superior performance for all soil attributes concerning the number of covariates (grid systems) used in the modeling, while Earth had the worst (Fig 14). With the increase in the number of covariates, there was a trend towards stabilizing performance. This demonstrates that the increase in the number of covariates used in modeling does not always improve performance [42,99]. There is a limit on maximum performance values, with increments in the number of covariates beyond which performance tends to stabilize or converge.

### Spatialization methods in perspective: comparing machine learning and ordinary kriging

Machine learning algorithms obtained the best prediction and spatialization performances when compared to IDW and KO. This is because the classical statistical methods for spatialization by IDW and OK reached a maximum optimization to be used automatically, in other words, they already are in their maximum performances for estimating spatialization [55,56]. On the other hand, the performance of ML algorithms can be improved with the addition of a new set of covariates. Only with information from the study area (limit and pixel size) was it possible to create grid systems, which could be used as new covariates in ML algorithms, but could be used in IDW and OK. As a result, there was an improvement in the prediction performance and spatialization of soil attributes when ML algorithms were used about IDW and OK. In this sense, ML algorithms can operate with a large number of covariates, which can increase the performance of the models, that is, they have not yet reached their limit to obtain maximum performance.

New geotechnologies such as remote and near sensing have emerged and can be used in soil science [100–102]. For instance, these technologies allow to creating soil and vegetation indices derived from sentinel 2 and Landsat 7 satellite images [103–106], which can be used as input data to improve the model’s performance in ML. In addition, it is possible to obtain and create more than 40 relief indices from open-source programs such as Saga Gis and Grass Gis directly and/or associated with other software such as R [103,107–109], which also can be used to improve the performance of the algorithm in ML. In this sense, the claim of lack of data to be used as input data in ML algorithms is not justifiable. Therefore, it is practically impossible to disregard ML techniques for the prediction and spatialization of soil attributes.

The use of ML also has three advantages over the classic IDW and OK methods: *i)* spatialization of categorical data (classification); *ii)* use of categorical covariates as input data in algorithms; *iii)* be able to mathematically obtain the importance of each covariate in the final prediction model. This set of features together or separate can lead to more realistic models, optimized and better performances. As a result, this provides more information, closer to reality and is mathematically supported with more reliable results for the end-user. Although not used in this research, ML algorithms can calculate and demonstrate the importance of covariates used to predict and spatialize attributes. Consequently, there is one information that is impossible to obtain directly by the IDW and OK methods. Given above, ML algorithms are a potential tool to replace the classic interpolator methods performed by IDW and OK.

From a practical standpoint, the enhanced spatial accuracy and reduced uncertainty achieved by the Euclidean-grid ML framework have direct implications for agricultural and environmental management. More reliable maps of soil texture, phosphorus availability, and cation exchange capacity can support precision fertilization, irrigation scheduling, land suitability assessment, and conservation planning. By minimizing the propagation of spatial errors, this approach improves decision-making under conditions where traditional interpolators often yield misleading or overly smoothed predictions. Thus, the proposed methodology not only advances digital soil mapping theory but also provides tangible benefits to applied soil science and agricultural practice.

## Conclusions

Machine learning generally demonstrates superior or comparable performance to traditional geostatistical methods for most soil attributes evaluated, even when relying exclusively on covariates derived from Euclidean grid systems. Across all attributes, Random Forest models combined with grid-based Euclidean covariates achieved the highest predictive accuracy, with R^2^ values reaching up to 0.70 for sand, 0.68 for clay, 0.62 for phosphorus, and 0.51 for CEC. These values represent clear improvements relative to Ordinary Kriging and Inverse Distance Weighting, which consistently produced lower R^2^, higher errors, and greater prediction uncertainty. These results demonstrate that machine learning can serve as an effective interpolator and, when coupled with simple spatial covariates, can outperform classical geostatistical approaches.

An important contribution of this study is the explicit use of Euclidean distance–based grid covariates to incorporate spatial structure into machine learning models. This approach differs from traditional DSM methods by enabling spatial prediction using only geometric information from the study area, without requiring environmental covariates or variogram modeling. The consistent improvements over OK, IDW, and baseline ML models demonstrate that Euclidean-grid spatial encoding is an effective and innovative strategy for enhancing soil attribute prediction.

Importantly, the proposed Euclidean-grid machine learning framework was rigorously benchmarked against established baseline methods, including OK, IDW, and standalone ML models using only X-Y coordinates. The consistent gains in accuracy, lower RMSE and MAE, and reduced spatial uncertainty confirm that the improvements observed arise directly from the explicit incorporation of Euclidean-based spatial covariates rather than from algorithmic differences alone. This establishes a clear methodological contribution to digital soil mapping by demonstrating that spatial structure can be effectively reconstructed without requiring environmental covariates or variogram modeling.

Our findings also show that algorithms based on multiple decision trees (e.g., Random Forest) deliver substantially better predictive performance than single-tree models such as Cubist. Among all evaluated techniques, Earth presented the weakest performance, while RF was consistently the most robust across attributes and grid configurations. Larger grid systems generally improved predictions but led to increased computational costs, highlighting an important trade-off between accuracy and processing time. The null model comparison further confirmed that all ML, OK, and IDW approaches provided meaningful predictive skill beyond the spatial average.

From a practical perspective, the enhanced accuracy and reduced uncertainty achieved through the Euclidean-grid ML framework have direct implications for agricultural management and environmental monitoring. More reliable maps of soil texture, phosphorus availability, and cation exchange capacity can support precision fertilization, soil conservation planning, land suitability assessment, and decision-making in data-scarce environments. Because the method requires only spatial coordinates and grid-derived Euclidean distances, it can be applied even where auxiliary covariates such as remote sensing data or digital terrain models are unavailable.

Overall, this study demonstrates that integrating Euclidean grid-based covariates into machine learning models constitutes a scalable, flexible, and computationally feasible alternative to classical geostatistical interpolators. The methodological advances presented here contribute to ongoing efforts to improve digital soil mapping workflows and provide robust, practical tools for soil science, agriculture, and land-use management.
